# Gastric schwannoma: report of two cases and review of the literature

**DOI:** 10.1016/j.ijscr.2018.10.062

**Published:** 2018-11-03

**Authors:** Daniel Paramythiotis, Anestis Karakatsanis, Diamantoula Pagkou, Petros Bangeas, Niki Mantha, Sofia Lypiridou, Antonis Michalopoulos

**Affiliations:** a 1st Propaedeutic Surgery Department, AHEPA University Hospital of Thessaloniki, Greece; b Pathology Department, Faculty of Medicine, Aristotle University of Thessaloniki, Greece

**Keywords:** Gastric schwannomas, Submucosal, Gist, Neoplasms

## Abstract

**Introduction:**

Gastrointestinal schwannomas are benign, slow-growing and usually asymptomatic tumors. In some cases bleeding, epigastric pain and palpable mass may be occurring. Preoperative diagnosis is challenging due to the difficulty of differentiation from other submucosal tumors. Diagnosis is most often provided through the histology report.

**Case presentation:**

In this study we report two cases of gastric tumors with the suspicion of a GIST preoperatively but histologically confirmed to be gastric schwannomas. Two patients of our study gave to us their written consent for publication. Research work has been reported with the PROCESS criteria.

**Discussion:**

Surgical resection should be considered the mainstay of treatment in patients with gastric schwannomas. Possible complications such as bleeding or pyloric stenosis can be presented. The size and location of the tumor, as well as its relation to the surrounding organs, are essential factors in determining the type of resection.

**Conclusion:**

Gastric schwannomas are usually presented us submucosal mass. Preoperative diagnosis is challenging due to the difficulty of differentiation from other submucosal tumors. Endoscopic Esophagogastroduodenoscopy with biopsy and endoscopic ultrasound is essential to determine the nature of these lesions. Resection of the lesion in healthy borders is the treatment of choice. Patho-logical examination usually revealed positive S-100 protein and negative CD34, CD117, Actin and desmin strains.

## Highlights

• Schwannomas are mesenchymal tumors.
• Schwannomas are benign, slow-growing and usually asymptomatic tumors, but in some cases bleeding, epigastric pain and palpable mass may occur.
• Preoperative diagnosis is challenging due to the difficulty of differentiation from other sub-mucosal tumors.
• The size and location of the tumor, as well as its relation to the surrounding organs, are essential factors in determining the type of operation.
• Local extirpation, wedge resection, partial, subtotal or even total gastrectomy, are all acceptable operations.
• Gastric schwannomas have a good prognosis.

## 1 Introduction

Mesenchymal tumors of the gastrointestinal tract represent a spectrum of spindle cell tumors that look similar under light microscopic examination, and these include gastrointestinal stromal tumors (GISTs), leiomyomas or leiomyosarcomas, and schwannomas [1]. Among these neoplasms, GISTs are the most common (60–70% of GISTs are occurring in the stomach), while schwannomas are neoplasms that only seldom arise in the gastrointestinal tract [2]. Gastrointestinal schwannomas are benign, slow-growing and usually asymptomatic tumors, but in some cases bleeding, epigastric pain and palpable mass may occur. Preoperative diagnosis is challenging due to the difficulty of differentiation from other submucosal tumors, and consequently, the correct diagnosis is most often provided through the histology report [3,4]. We report two cases of gastric tumors with the suspicion of a GIST preoperatively but histologically confirmed to be gastric schwannomas. Patients gave to us their written consent for publication. Our research has been reported according PROCESS criteria [5] with registration number researchregistry4205.

## 2 Case report 1

**Fig. 1:**
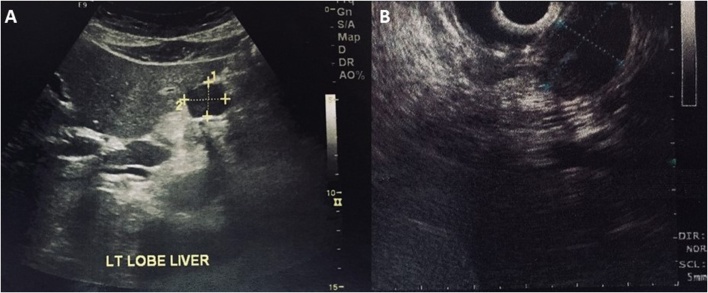
A: Ultrasonography scan of the upper abdomen shows a round and well-defined mass in the stomach. Fig. 1B: Endoscopic ultrasound reveals a 2.5 × 1.5 cm hypoechoic mass that appears to arise from the muscolaris propria of the lesser curvature.

**Fig. 2:**
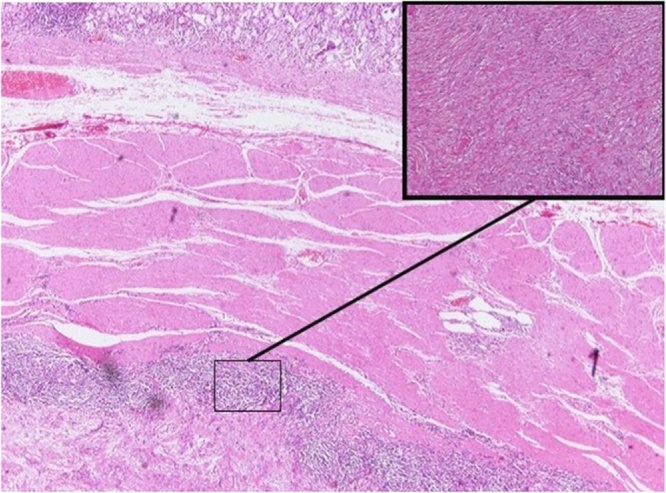
Spindle cells arranges in fascicles (HE 4×) and in the frame the mass partially surrounded by lymphoid aggregates (HE 10×).

**Fig. 3:**
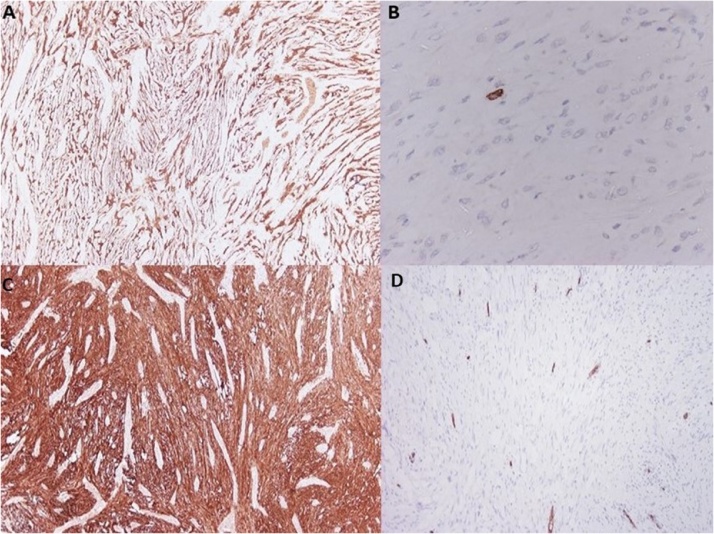
Tumor cells are positive for S-100 protein in both cases (IHC case 1, 4× Fig. 3A), (IHC case 2, 4× Fig. 3C) and negative for CD-117 protein (ICH case 1, 40× Fig. 3B) and CD-34 (IHC case 2, 10× Fig. 3D).

54-year old female with a history of hypertension and gallbladder polyp was admitted to our department for the evaluation of a gastric lesion that was detected incidentally during ultrasonography scan of the upper abdomen (Fig. 1A). Esophagogastroduodenoscopy (EGD) and endoscopic ultrasound (EUS) (Fig. 1B) confirmed the presence of a 2.5 × 1.5 cm hypoechoic and submucosal lesion situated along the lesser curvature of the stomach. Tumor markers of Alpha-fetoprotein, (AFP), Cancer Antigen 125 (CA-125), Cancer Antigen-19.9 (CA19.9), and Carcinoembryonic Antigen (CEA) were all within limits. A contrast-enhanced computed tomography (CT) scan revealed a homogeneous exophytic mass at the lesser curvature of the middle body of the stomach. No intra-hepatic lesions were seen, and the other abdominal organs were unremarkable. Endoscopic biopsy revealed submucosal mass contiguous with the muscularis propria. Chronic inflammation with fibro-sis were also detected. Surgical approach was decided, and the patient underwent an open cholecystectomy and local resection of the gastric mass in healthy borders. Macroscopic examination of the resected mass revealed a well-circumscribed nodular tumor measured 2.8 × 1.5 × 1.8 cm. Histopathology findings of frozen section was characterized by interlacing bundles of spindle cells of varying cellularity and peripheral lymphoid cuffs (Fig. 2). Neoplastic cells were strongly positive for S-100 protein (Fig. 3A), but they were negative for CD-34, CD-117 (Fig. 3B), smooth-muscle actin and desmin. The resected margin was reported clear (R0). Postoperative period was uneventful, and one-month follow-up was unremarkable.

## 3 Case report 2

**Fig. 4:**
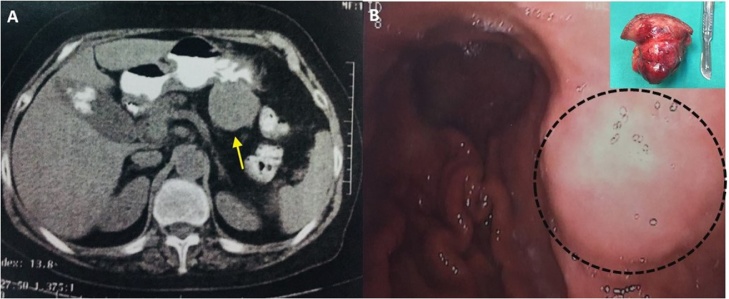
Contrast enhanced CT showing a round, well-defined and homogeneous gastric mass (Fig. 4A) and endoscopic detection (Fig. 4B) in submucosal of the greater curvature of the stomach. In the frame, macroscopic view of the resected mass shows a large exophytic mass along the less curvature of the stomach.

77-years old female with a history of cholelithiasis presented with epigastric pain persisting for the last six months. CT scanning revealed a homogeneous round mass measuring 5 cm, arising from the posterior wall of the stomach (Fig. 4A). Submucosal tumor with the possibility of GIST was suspected, and surgical intervention was recommended. Upper gastrointestinal endoscopic examination revealed a protruding submucosal mass between antrum and body of the stomach along greater curvature (Fig. 4B). The overlying mucosa was normal. Endoscopic biopsy revealed chronic inflammation without suspicious cells for malignancy. During abdominal exploration with midline excision, a 5 cm sized exophytic mass was identified in the posterior wall of the greater curvature be-tween body and antrum. There was no infiltration of the mass into the surrounding tissues, nor any distal metastasis in other organs. A tumor was excised, and combined cholecystectomy was per-formed due to cholelithiasis. Histopathology findings revealed a 5 cm well-circumscribed but not capsulated mass arising from muscular propria with intact overlying mucosa. A picture of spindle cells with areas of hypo and hypercellularity (Antoni A and Antoni B areas) with a peripheral cuff of peritumoral lymphoid aggregates were identified. Tumor cells showed strong immunoreactivity for S-100 protein (Fig. 4C) but were negative for CD-117 and CD-34 (Fig. 4D).

## 4 Discussion

Schwannomas, also known as neurilemmomas or neurinomas, are benign neurogenic tumors, originating from Schwann cells, which generally form the sheath around the axons of the peripheral nerves. Although schwannomas can develop anywhere along the peripheral course of the nerve, they most commonly occur in the head and neck but rarely in the GI tract, where, they arise from the nerve plexus of the gut wall (Auerbach’s, and less commonly Meissner plexus) [1,2]. The stomach is the most common site for schwannomas in the gastrointestinal tract, followed by the colon. The most infrequently affected sites are the small intestine and esophagus [3].

Schwannomas account 0.2% of all gastric tumors, 6.3% of gastric mesenchymal tumors, and 4% of all benign tumors of the stomach [3]. It has been reported that for each gastric schwannoma, there are approximately 45 gastric GISTs [2,3]. More than 221 cases of gastric schwannomas have been reported worldwide, and these may occur at any age. However, they are most frequently observed in the fifth and sixth decade of life with a variable female predominance (male/females 1:2,5) [1,3]. Most authors agree that a significant proportion (around 40%) of gastric schwannomas are asymptomatic [1,3,5], discovered incidentally on cross-sectional imaging or during endoscopy. The most common presenting symptom is abdominal pain or discomfort, followed by upper GI bleeding. Bleeding may be secondary to the growing submucosal mass compromising the blood supply to the overlying mucosa, or from a reduced tolerance to the gastric acidity. Other less frequent symptoms include poor appetite, dyspepsia, weight loss and nausea or vomiting [2,3,5,6]. CT, MRI, and EUS of upper gastrointestinal, remaining as complimentary imaging studies in patients presenting without specific findings and the definitive diagnosis of gastric schwannomas is determined on the pathology report. Gastric schwannomas are uniquely different from other schwannomas in that they show similar attenuation on CT scans and that degenerative changes such as cystic changes are uncommon [7]. Furthermore, this homogenous enhancement pattern may also aid differentiating between gastric schwannomas and GISTs, since GISTs frequently show heterogeneous enhancement due to degenerative changes [7]. MRI may provide further information about the exact location of the tumor and its relation to the surrounding structures. Schwannomas appear on MRI as strongly enhancing tumors, having low to medium signal intensity on T1 weighted images and high signal intensity on T2 weighted sequences [8]. EUS is useful for visualizing the submucosal lesions of the stomach, while the endosonographic features of gastric schwannomas include homogeneous hypoechoic internal echoes with a marginal halo [8]. Endoscopy apart from revealing the exact location of the gastric schwannoma, which is usually located in the middle third of the stomach along the lesser curvature [2], demonstrate that most of these tumors are found to be encased by intact mucosa. Principally, they involve the submucosa and muscularis propria, without invading adjacent structures and about half of them show central ulceration [5,6]. Though endoscopic needle biopsy is useful in establishing a definite diagnosis of a submucosal tumor, in case the mass proves to be a GIST, there is the risk of bleeding or rupture of the tumor which is associated with poor prognosis. Therefore, needle biopsy is not usually advocated [6]. Typical histologic features that are valuable in recognizing gastric schwannomas are the presence of focally atypical spindle cells, which are typically arranged in a microtrabecular-microvascular pattern, and a peritumoral lymphoid cuff, often with germinal centers. Immuno-histochemical studies show universal positivity for S100 protein and frequent, albeit variable, immunoreactivity for glial fibrillary acidic protein (GFAP) and CD56. Scattered epithelial membrane antigen (EMA) positivity may be seen in peri-neural cells, while these gastric schwannomas are negative for CD117, DOG-1, CD34, muscle actin and desmin. These morphologic and immunohistochemical findings are confirmatory of the diagnosis [3,9]. Gastric schwannomas variated histologically from soft tissue schwannomas. Gastric schwannomas, origin from the dispersed autonomic nerve Schwann cells, as opposed to being encased by epineurium, as in the case of soft tissue schwannomas. Additionally, nuclear palisading, xanthomatous cells, and vascular hyalinization and dilatation, are rare in gastric schwannomas, in contrast to their soft tissue counterparts. On the other hand, prominent lymphoid infiltration, micro trabecular architecture and frequent nuclear atypia are not features of soft tissue schwannomas [4,9]. To prevent possible complications such as bleeding or pyloric stenosis, surgical resection should be considered the mainstay of treatment in patients with gastric schwannomas. The size and location of the tumor, as well as its relation to the surrounding organs, are essential factors in determining the type of operation. In addition to these, the absent malignant potential is also an important factor that should be taken into consideration when determining the extent of surgery. Therefore, no lymphadenectomy is warranted [9]. Even though there are publications that report malignant gastric schwannomas, it has been proposed that these reports present tumors with characteristics slightly more pertinent to GISTs than to schwannomas [3,4,9]. Therefore, local extirpation, wedge resection, partial, subtotal or even total gastrectomy, are all acceptable operations while laparoscopic techniques may also be used, provided that the equipment, as well as the technical expertise, is available [6]. Since the recurrent disease is generally associated only with incomplete surgical mar-gins, gastric schwannomas have a good prognosis [4,9,10]. The only concern is about gastric schwannomas with mitotic rates higher than 10/50 HPF, due to limited experience on their long-term follow-up [4,10]. Presence of H. pylori in the extracted specimen, as found in one of our patients, has only recently been presented in another case report [11]. Despite the fact that the role of microorganism has been well established in the pathogenesis of gastric adenocarcinoma, in case of gastric schwannomas the correlation is uncertain [12,13].

## 5 Conclusion

Schwannomas are benign submucosal lesions, originating from Schwan cells. They most commonly occur in the head and neck but rarely they arise from nerve plexus of GI wall (Auerbach and Meissner plexus). Stomach is the most common site followed by the colon. Preoperative diagnosis is challenging due to the difficulty of differentiation from other submucosal tumors. The most common presenting symptom is abdominal pain or discomfort, followed by upper GI bleeding. CT, MRI, and EUS of upper gastrointestinal, were usually without specific findings and the definitive diagnosis of gastric schwannomas is determined on final pathology report. Resection of the lesion in healthy borders is the treatment of choice. Immuno-histochemical studies show universal positivity for S100 protein and frequent negative for CD117, DOG-1, CD34, muscle actin and desmin strains.

## Conflicts of interest

No conflict of interest.

## Sources of funding

This study is own funding.

## Ethical approval

We have approval from bioethical committee of Aristotle University of Thessaloniki Greece.

## Consent

Written informed consent was obtained from the patient for publications of this case report and accompanying images. A copy of the written consent is available for review by the Editor-in-Chief of this journal on request.

## Author contribution

Daniel Paramythiotis for study design.

Anestis Karakatsanis writing the paper.

Diamantoula Pagkou, Niki Mantha and Sofia Lypiridou data collection.

Petros Bangeas writing the paper and data analysis.

Antonis Michalopoulos Study concept.

## Registration of research studies

researchregistry4205.

## Guarantor

Daniel Paramythiotis.

## Provenance and peer review

Not commissioned, externally peer reviewed.
